# Comparison of Classification Algorithms with Wrapper-Based Feature Selection for Predicting Osteoporosis Outcome Based on Genetic Factors in a Taiwanese Women Population

**DOI:** 10.1155/2013/850735

**Published:** 2013-01-14

**Authors:** Hsueh-Wei Chang, Yu-Hsien Chiu, Hao-Yun Kao, Cheng-Hong Yang, Wen-Hsien Ho

**Affiliations:** ^1^Department of Biomedical Science and Environmental Biology, Graduate Institute of Natural Products, College of Pharmacy, Cancer Center, Kaohsiung Medical University Hospital, Kaohsiung Medical University, Kaohsiung 807, Taiwan; ^2^Department of Healthcare Administration and Medical Informatics, Kaohsiung Medical University, Kaohsiung 807, Taiwan; ^3^Department of Electronic Engineering, National Kaohsiung University of Applied Sciences, Kaohsiung 807, Taiwan

## Abstract

An essential task in a genomic analysis of a human disease is limiting the number of strongly associated genes when studying susceptibility to the disease. The goal of this study was to compare computational tools with and without feature selection for predicting osteoporosis outcome in Taiwanese women based on genetic factors such as single nucleotide polymorphisms (SNPs). To elucidate relationships between osteoporosis and SNPs in this population, three classification algorithms were applied: multilayer feedforward neural network (MFNN), naive Bayes, and logistic regression. A wrapper-based feature selection method was also used to identify a subset of major SNPs. Experimental results showed that the MFNN model with the wrapper-based approach was the best predictive model for inferring disease susceptibility based on the complex relationship between osteoporosis and SNPs in Taiwanese women. The findings suggest that patients and doctors can use the proposed tool to enhance decision making based on clinical factors such as SNP genotyping data.

## 1. Introduction

The World Health Organization (WHO) has defined osteoporosis as a skeletal disorder characterized by diminished bone strength resulting in increased fracture risk [1]. Bone strength is determined by interacting somatic and genetic factors [2]. Reported somatic factors include aging [2–5], menopause [5, 6], and body mass index (BMI) [4, 5, 7, 8]. To identify the genetic determinants of osteoporosis, an earlier study by the first author of this paper [9] investigated how the incidence of low bone mineral density (BMD) in Taiwanese women is affected by interactions among eleven single nucleotide polymorphisms (SNPs) in nine genes known to be involved in osteoporosis [10–16], including tumor necrosis factor-alpha (TNF*α*), transforming growth factor-beta 1 (TGFB1; TGF*β*1), Osteocalcin, parathyroid hormone (PTH), interleukin 1 receptor antagonist (IL1_ra), heat shock 70 kDa protein 1-like (HSPA1L; HSP70 hom), heat shock 70 kDa protein 1B (HSPA1B; HSP70-2), calcitonin receptor (CTR), and bone morphogenetic protein-4 (BMP-4). Generally, several hormones, cytokines, and cell signaling-related proteins were chosen. For example, CTR, which is a receptor for the linear polypeptide hormone calcitonin, reduces blood calcium and suppresses the effects of PTH [17]. The hormonal function of osteocalcin is to release insulin from the pancreas [18]. The cytokine family includes TNF*α*, TGF-*β*, BMP4 (protein of TGF-*β*superfamily), and IL-1RA (protein of interleukin 1cytokinefamily) whereas cell-signaling proteins include HSP70 hom and HSP70-2. Studies of the interactions among these hormones (e.g., [9] and references therein) indicate that osteoporosis is an endocrinological problem.

Several gene polymorphisms may cooperatively contribute to the development of osteoporosis in Taiwanese women. Accumulating evidence reveals that SNPs are potential genetic markers for predicting osteoporosis outcome in Taiwanese women [9]. Chang et al. [19] also proposed a novel odds ratio-based genetic algorithm (OR-GA) method of using odds ratios for quantitatively measuring the disease risk associated with various SNP combinations to determine the susceptibility to osteoporosis in Taiwanese women. Taiwanese women who are carriers of risk alleles in two or more of these SNPs are likely to be at increased risk of osteoporosis because several partial deficiencies in these pathways may severely diminish bone density. Therefore, SNPs may indicate risk of osteoporosis in Taiwanese women and may be useful in clinical association studies to determine the genetic basis of disease susceptibility.

The risk of osteoporosis is likely to be higher than normal in carriers of risk alleles in two or more of these SNPs because several partial deficiencies in these pathways may substantially decrease bone density. Therefore, interacting polymorphisms may affect osteoporosis risk. In [9], the effects of age, BMI, and genetic factors on BMD were evaluated in pre- and postmenopausal Taiwanese women were evaluated. Eleven interacting polymorphisms in nine genes were studied in terms of their effects on the incidence of low BMD (Table 1). Combinations of SNPs were evaluated for genotype associations in women with osteoporosis. The findings showed that specific SNP combinations may be risk factors for postmenopausal osteoporosis in Taiwanese women. In addition to these specific SNP combinations, BMI and age also showed independent associations with BMD in postmenopausal Taiwanese women.

Although an apparent association between SNPs and osteoporosis has been identified in Taiwanese women, a continuing challenge in genomics studies of Taiwanese women populations lies in identifying significant genes. Exhaustive computation over the model space is infeasible if the model space is very large, as there are 2^p^ models with p SNPs [20, 21]. Feature selection techniques are designed to find responsible genes and SNPs for certain diseases. By selecting a small number of SNPs with significantly larger effects compared to other SNPs and by disregarding SNPs of lesser significance, researchers can focus on the most promising candidate genes and SNPs for use in diagnosis and therapy [21, 22].

In [9], combined polymorphisms in different genomic regions were evaluated for associations with BMD variation. The findings showed that a combination of several gene polymorphisms contributes to the development of osteoporosis in Taiwanese women. However, that study did not report a subset of SNPs that can be used to predict osteoporosis outcome in this population. Therefore, the current study used the same dataset used in [9] to elucidate the relationship between osteoporosis and SNPs in Taiwanese women in a performance comparison of three different classification algorithms with wrapper-based feature selection [23]: multilayer feedforward neural network (MFNN) [24–28], naive Bayes [29], and logistic regression [30]. The MFNNs have proven particularly effective for nonlinear mapping based on human knowledge and are now attracting interest for use in solving complex classification problems [24]. An MFNN containing layers of simple computing nodes, which is analogous to brain neural networks, has proven effective for approximating nonlinear continuous functions and for revealing previously unknown relationships between given input and output variables [25, 26]. The unique structure of MFNNs enables them to learn by using algorithms such as backpropagation and evolutionary algorithms [31, 32]. Potential medical applications of MFNNs include solving problems in which the relationship between independent variables and clinical outcome are poorly understood [33]. Because MFNNs are capable of self-training with minimal human intervention, many studies of large epidemiology databases have, in addition to conventional statistical methods, used MFNNs for further insight into the interrelationships among variables. A naive Bayes classifier assumes that the presence (or absence) of a particular feature of a class is unrelated to the presence (or absence) of any other feature, given the class variable. Depending on the precise nature of the probability model, naive Bayes classifiers can be trained very efficiently in a supervised learning setting. The classifier obtained by using this set of discriminant functions and by estimating the relevant probabilities from the training set is often called the naive Bayesian classifier because, if the the attributes are “naively” assumed to be independent given the class, direct application of the Bayes theorem easily confirms that this classifier is optimal in terms of minimizing the misclassification rate or zero-one loss [34, 35]. Logistic regression is a statistical method of predicting the outcome of a variable that is categorical (i.e., it can have several different categories) and is dependent on one or more predictor variables. A logistic function can be used to model the probabilities describing the possible outcome of a single trial as a function of explanatory variables. Logistic regression is typically used to measure the relationship between a categorical dependent variable and one or more continuous independent variables by converting the dependent variable to probability scores [36].

The wrapper-based feature selection method [23], in which the feature selection algorithm acts as a wrapper around the classification algorithm, was also used to identify an SNP subset with sufficient predictive power to distinguish between high- and low-risk alleles. In the wrapper-based approach, the function used to evaluate feature subsets uses the classification algorithm itself to perform a best-first search for a good subset [23]. Starting from an empty feature set, it searches forward for potential feature subsets by performing greedy hillclimbing augmented with a backtracking technique [37]. The wrapper-based feature selection method is applied here because Huang et al. [21] showed that it may be superior to hybrid approaches combining chi-square and information-gain methods reported in the literature. A comprehensive literature review shows no attempts to predict osteoporosis outcome in Taiwanese women using genetic factors (SNPs) and the three above mentioned classification algorithms with wrapper-based feature selection method.

This study therefore compared performance in three classification algorithms: MFNN, naive Bayes, and logistic regression, with and without wrapper-based feature selection techniques. Identifying the genes and SNPs associated with Taiwan population of women with osteoporosis would enable researchers to focus on the candidate genes and SNPs that are most promising for use in diagnosis and therapy. The results of our studies could be generalized to SNP searches in genetic studies of human disorders and to development of new molecular diagnostic/prognostic tools. However, before routine application of genomic analysis in clinical practice, genetic markers must be validated in prospective clinical trials.

## 2. Materials and Methods

### 2.1. Subjects

The dataset in this study, which included SNPs, age, menopause, and BMI, was the same dataset used in a previous study by the first author of this paper [9]. The *T*-score was calculated according to WHO classifications using a locally derived reference range provided by the manufacturer. The subjects were divided into two BMD groups according to *T*-score [38–40]. Subjects with *T*-score > −1 were enrolled in the high BMD group, and those with *T*-scores ≤ −1 were enrolled in the low BMD group. The overall dataset was derived from 295 cases, including (i) 247 postmenopausal cases (83.73%) and 48 prepremenopausal cases (16.27%); (ii) 112 high BMD cases (37.97%) and 183 low BMD cases (62.03%).  Table 2 presents the demographic characteristics of the study subjects. Post-menopause was defined as the absence of menstruation for >6 months or age ≥ 50 years [9]. Clinical data used for diagnosis were further converted into numerical form, that is, 1 for “high BMD” and 0 for “low BMD.”

### 2.2. Candidate Genes

Table 1 shows the 22 SNPs analyzed in this study, which were the same as those analyzed previously by the first author of this paper [9]. Table 1 shows that the nine candidate genes included TNF*α*, transforming growth factor-beta 1 (TGF*β*1), osteocalcin, parathyroid hormone (PTH), interleukin 1 receptor antagonist (IL1_ra), HSP, calcitonin receptor (CTR), bone morphogenetic protein-4 (BMP-4), and three genotypes per locus.

### 2.3. Classification Algorithms

The three families of classification algorithms used as the basis for comparisons in this study were MFNN, naive Bayes, and logistic regression. These classifiers were implemented using the Waikato Environment for Knowledge Analysis (WEKA) software [37]. 

An MFNN is an artificial neural network (ANN) model in which connections between the units do not form a directed cycle [24–28, 30]. From an algorithmic perspective, the underlying process of an MFNN can be divided into retrieving and learning phases [24]. Assume an *L*-layer feedforward neural network with *N*_*l*_ units at the *l*th layer. In the retrieving phase, the MFNN iterates through all layers to produce the retrieval response {*a*_*i*_(*L*), *i* = 1, 2,…, *N*_*L*_} at the output layer based on test pattern inputs {*a*_*i*_(0), *i* = 1, 2,…, *N*_0_}, the known weights *w*_*ij*_ of the network, and the nonlinear activation function *f*_*i*_ (e.g., sigmoid function). In the learning phase of this MFNN, the backpropagation algorithm [30] and evolutionary algorithms [31, 32] are used in the learning scheme. The backpropagation algorithm is used as a simple gradient descent approach. The weight updating mechanism is a backpropagation of corrective signals from the output layer to the hidden layers. The goal is iteratively selecting a set of weights *w*_*ij*_(*l*) for all layers such that the squared error function *E* can be minimized by a pair of input training patterns {*a*_*i*_(0), *i* = 1, 2,…, *N*_0_} and target training patterns {*t*_*j*_, *j* = 1, 2,…, *N*_*L*_}. 

Mathematically, the iterative gradient descent formulation for updating each specific weight *w*_*ij*_(*l*) can be expressed by the following equation:

(1) wij(l)⟵wij(l)−η∂E∂wij(l),

where *η* is the learning rate and ∂*E*/∂*w*_*ij*_(*l*) can be effectively calculated through a numerical chain rule by backpropagating the error signal from the output layer to the input layer.

Structurally, however, an MFNN is a spatial and iterative neural network with several layers of hidden neuron units between the input and output neuron layers. The basic function of each neuron is the linear basis function, and activation is modeled with a non-decreasing and differentiable sigmoid function. This approach uses an MFNN to model osteoporosis outcome. Inputs contain the information about clinical factors, for example, SNPs, that are needed for the database. Outputs contain the information about the osteoporosis outcome. 

In summary, the MFNN is trained first by repeatedly providing input-output training pairs and by executing the backpropagation learning algorithm. After this training process is complete, the MFNN is tested by sending testing data inputs (i.e., SNPs) to the network. The forward propagation of the MFNN reveals the osteoporosis outcome for a specific case so that causes can be inferred from effects. Here, the default WEKA parameters were used, that is, hidden layer neurons = 6, learning rate = 0.3, momentum variable = 0.2, and training time = 500.

Second, all features in naive Bayes, which is the simplest Bayesian network, are assumed to be conditionally independent [34]. Let (*X*_1_, *X*_2_,…, *X*_*p*_) be features (i.e., SNPs) used to predict class *C* (i.e., disease status, 1 = high BMD or 0 = low BMD). Given a data instance with genotype (*x*_1_, *x*_2_,…, *x*_*p*_), the best prediction of the disease class is given by class *c*, which maximizes the conditional probability Pr(*C* = *c* | *X*_1_ = *x*_1_, *X*_2_ = *x*_2_,…, *X*_*p*_ = *x*_*p*_). Bayes theorem is used to estimate the conditional probability Pr(*C* = *c* | *X*_1_ = *x*_1_, *X*_2_ = *x*_2_,…, *X*_*p*_ = *x*_*p*_), which is decomposed into a product of conditional probabilities. 

Third, the logistic regression generates the coefficients for the following formula used for logit transformation of the probability of a patient having a characteristic of interest: logit(*p*) = *b*_0_ + *b*_1_ *x*_1_ + *b*_2_ *x*_2_ + ⋯+*b*_*k*_ *x*_*k*_ [41]. The formula used to calculate the probability of the characteristic of interest in this study is *p* = 1/(1 + *e* ^−logit(*p*)^), where 1 = high BMD and 0 = low BMD.

### 2.4. Feature Selection

**Figure 1:**
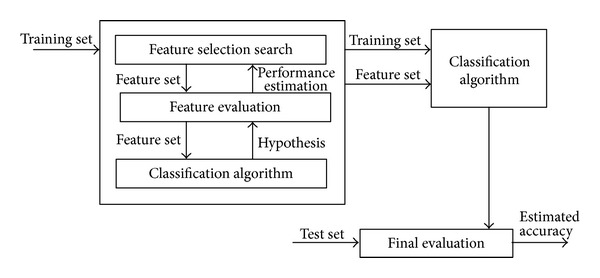
Flowchart of wrapper-based approach to feature subset selection [23].

**Figure 2:**
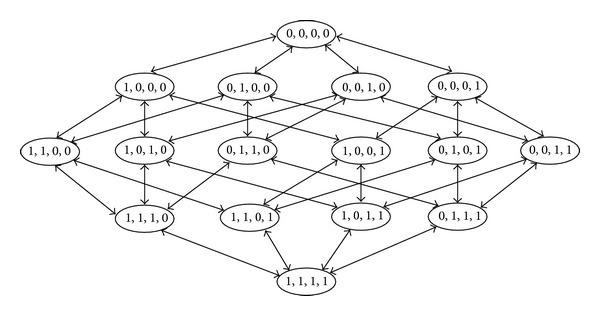
State space search for feature subset selection [23].

The wrapper-based feature selection approach [23], in which a feature selection algorithm acts as a wrapper around a classification algorithm, was used to find a subset of SNPs that maximizes the performance of the prediction model.  Figure 1 shows that, in the wrapper approach, the feature subset is selected by using a black box classification algorithm (i.e., selection is performed using the interface alone and does not require knowledge of the algorithm). To search for a good subset, the feature subset selection algorithm includes the classification algorithm itself in the evaluation function. The accuracy of the deduced classifiers is estimated using accuracy estimation techniques. The search space is organized such that each state represents a feature subset. For *n* features, each state has *n* bits, and each bit indicates whether a feature is present (1) or absent (0). To determine the connectivity between the states, this study used operators that add or delete a single feature from each state, where the states correspond to the search space commonly used in stepwise method [23].  Figure 2 shows an example of the state space and operators obtained by stepwise method in a four-feature problem. The size of the search space for *n* features is O(2^*n*^) [23]. The classification algorithms are used to calculate a performance measure for each of 16 different subsets. 

**Figure 3:**
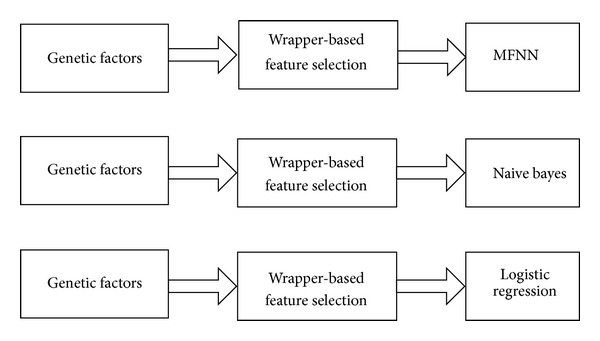
In the wrapper-based feature selection approach, genetic factors are evaluated independently of multilayer feedforward neural network (MFNN), naive Bayes, and logistic regression.

Therefore, the wrapper-based approach conducts a best-first search for a good subset by including the classification algorithm itself (MFNN, naive Bayes, or logistic regression) in the feature subset evaluation [23]. To search for potential feature subsets, the best-first search starts from an empty feature set and searches forward by greedy hillclimbing augmented with a backtracking technique [37].  Figure 3 shows how MFNN, naive Bayes, and logistic regression were applied in the wrapper-based approach. 

### 2.5. Evaluating Predictive Performance

The performance of the prediction models was measured in terms of receiver operating characteristic (ROC) and area under the ROC curve (AUC) [42]. The AUC of a classifier can be interpreted as the probability of the classifier ranking a randomly chosen positive example higher than a randomly chosen negative one [42]. Most researchers have now adopted AUC for evaluating the predictive capability of classifiers since AUC is a better performance metric compared to accuracy [42]. This study used the AUC value for performance comparison of different prediction models using the same dataset. The higher the AUC, the better the learning performance [43]. Other calculations included sensitivity, the proportion of correctly predicted responders out of all tested responders, and specificity, the proportion of correctly predicted nonresponders out of all tested nonresponders.

To investigate the generalization of the prediction models produced by the above algorithms, the repeated 10-fold cross-validation method was used [44]. First, the whole dataset was randomly divided into ten distinct parts. The model was then trained with nine-tenths of the data and tested by the remaining tenth of data to estimate its predictive performance. This procedure was repeated nine more times. Each time, a different tenth of the data was used as testing data, and a different nine-tenths of the data were used as training data. Finally, the average estimate over all runs was reported by running the above regular 10-fold cross-validation 100 times with different splits of data. In repeated 10-fold cross-validation testing, the performance of all models was evaluated with and without feature selection.

## 3. Results

Tables 3 and 4 summarize the results of the repeated 10-fold cross-validation experiments for MFNN, naive Bayes, and logistic regression using SNPs with and without feature selection. First, the AUC, sensitivity, and specificity were calculated for the three predictive models without wrapper-based feature selection.  Table 3 shows that the average AUC values for the MFNN, the naive Bayes, and the logistic regression prediction models were 0.489, 0.462 and 0.485, respectively. In terms of AUC, the the MFNN model (AUC = 0.489) outperformed the naive Bayes (AUC = 0.462) and logistic regression (AUC = 0.485) models. 

A repeated 10-fold cross-validation experiment was performed to compare performance in the three wrapper-based predictive algorithms.  Table 4 shows that the MFNN, the naive Bayes, and the logistic regression models had average AUC values of 0.631, 0.569, and 0.620, respectively. In terms of AUC, the MFNN model (AUC = 0.631) outperformed both the naive Bayes model (AUC = 0.569) and the logistic regression model (AUC = 0.620). Each wrapper-based model selected 3 to 8 SNPs (Table 4). Out of 11 SNPs, the wrapper-based MFNN model identified only 4: rs1800469 (TGF*β*1-509), VNTR (IL1_ra), rs2227956 (HSP70 hom), and rs1801197 (CTR). 

The classifiers were also compared with and without feature selection. Feature selection using the wrapper-based approach clearly improved performance in the MFNN, the naive Bayes, and the logistic regression. Overall, the MFNN classifier with the wrapper-based approach demonstrated superior prediction performance (AUC = 0.631) compared to the other models. Additionally, the MFNN classifier with wrapper-based feature selection required fewer SNPs (*n* = 4) compared to the MFNN classifier without feature selection (*n* = 11).

Table 4 shows that the AUCs did not significantly differ between the MFNN model with wrapper-based feature selection (AUC = 0.631) and the logistic regression model with wrapper-based feature selection (AUC = 0.620). However, the MFNN classifier with wrapper-based feature selection required fewer SNPs (*n* = 4) compared to the logistic regression classifier with wrapper-based feature selection (*n* = 8), that is, by selecting a small number of SNPs with significantly larger effects compared to other SNPs and by disregarding relatively insignificant SNPs, the MFNN model with wrapper-based feature selection successfully identified a subset of four major SNPs that could be used to predict osteoporosis outcome in the study population (rs1800469 (TGF*β*1-509), VNTR (IL1_ra), rs2227956 (HSP70 hom), and rs1801197 (CTR)). After confirming that the MFNN model outperforms the logistic regression model, the next objective was finding the candidate genes and SNPs that are most promising for diagnosing osteoporosis, designing therapies, and predicting outcome in the studied population of Taiwanese women with osteoporosis.

## 4. Discussion

This study compared three classification algorithms, including MFNN, naive Bayes, and logistic regression with and without feature selection in terms of accuracy in predicting osteoporosis outcome in a population of Taiwanese women. Accounting for models is not a trivial task because even a relatively small set of candidate genes obtains a large number of possible models [20]. For example, the 11 candidate SNPs studied yielded 2^11^ possible models. The three classifiers were chosen for comparison because they cover varying techniques with different representational models such as probabilistic MFNN, naive Bayes, and logistic regression models [43]. The proposed procedures can also be implemented using the publicly available software WEKA [37] and are thus easily applicable in genomic studies. To the best of our knowledge, this study is the first to propose the use of three classification algorithms, including MFNN, naive Bayes, and logistic regression, and wrapper-based feature selection method for modeling osteoporosis outcome in Taiwanese women based on genetic factors such as SNPs.

In this paper, the wrapper-based feature selection approach was used to find a subset of SNPs that maximizes the performance of the prediction model according to how feature selection search is incorporated in the classification algorithms. The results showed that the MFNN classifier with wrapper-based approach was superior to the other tested algorithms and achieved the greatest AUC with the smallest number of SNPs when distinguishing between high and low BMD in Taiwanese women. These results suggest that MFNN model is a good method of modeling complex nonlinear relationships among clinical factors and the responsiveness of osteoporosis outcome in Taiwanese women. The wrapper-based approach does not require knowledge of the classification algorithm used in the feature selection process, in which features are optimized by using the classification algorithm as part of the evaluation function [21, 23]. Another advantage of the wrapper-based method is its inclusion of the interaction between feature subset search and the classification model [21]. However, the risk of over-fitting is high when using the wrapper-based method [21, 45]. In the current study, use of the wrapper-based feature selection approach to assess high and low BMD individuals revealed a panel of genetic markers, including TGF*β*1-509, IL1_ra, HSP70 hom, and CTR, which were more prominent compared to other markers observed in the examined Taiwanese women population with osteoporosis. 

A noted limitation of this study is that, due to the small sample size, the AUC values were too low (<0.7) to obtain good dataset classifications. A dataset based on a larger sample size is needed for improved accuracy. Therefore, further prospective clinical trials are recommended to determine whether the observed outcome associations with these candidate genes are reproducible in a larger population of Taiwanese women with osteoporosis.

## 5. Conclusion

This study used an MFNN methodology with wrapper-based feature selection method to predict osteoporosis outcome in Taiwanese women based on clinical factors such as SNPs. The trained MFNN model showed good responsiveness in inferring osteoporosis outcome. The findings suggest that patients and doctors can use the proposed tool to enhance decision making based on clinical factors such as SNP genotyping data. However, genetic markers require validation in further prospective clinical trials before routine clinical use of genomic analysis for predicting osteoporosis outcome.

## Figures and Tables

**Table 1:** Panel of 11 SNPs [9].

SNP	Gene	rs number	Genotype		
			1	2	3
1	TNF*α*-857	rs1799724	TT	TC	CC
2	TGF*β*1-509	rs1800469	TT	TC	CC
3	Osteocalcin	rs1800247	CC	CT	TT
4	TNF*α*-308	rs1800629	AA	AG	GG
5	PTH (BstB I)	rs6254	GG	AG	AA
6	PTH (Dra II)	rs6256	AA	AC	CC
7	IL1_ra^b^	VNTR^a^	A1A1^b^	A1A2	A1A4
8	HSP70 hom	rs2227956	CC	CT	TT
9	HSP 70-2	rs1061581	GG	AG	AA
10	CTR	rs1801197	CC	CT	TT
11	BMP-4	rs17563	CC	CT	TT

^a^VNTR: various number of tandem repeat.

^b^IL1_ra genotype: A1: 410 bp; A2: 240 bp; A4: 325 bp.

**Table 2:** Demographic data for study subjects.

Factor	Range	Descriptive statistics
Age (year)	27–83	*μ* = 56.38; *σ* = 10.37
Menopause	Postpremenopausal/ Prepremenopausal	247 (83.73%)/48 (16.27%)
BMI (kg/m^2^)	17.22–35.49	*μ* = 23.53; *σ* = 2.874
BMD	High/low	112 (37.97%)/183 (62.03%)

BMI: body mass index; BMD: bone mineral density.

**Table 3:** Results of repeated 10-fold cross-validation experiment using multilayer feedforward neural network (MFNN), naive Bayes, and logistic regression without feature selection.

Algorithm	AUC	Sensitivity	Specificity	Number of SNPs
MFNN	0.489	0.400	0.629	11
Naive Bayes	0.462	0.296	0.612	11
Logistic regression	0.485	0.333	0.615	11

AUC: area under the ROC curve.

**Table 4:** Results of repeated 10-fold cross-validation experiment using multilayer feedforward neural network (MFNN), naive Bayes, and logistic regression with wrapper-based feature selection approach.

Algorithm	AUC	Sensitivity	Specificity	Number of SNPs
MFNN	0.631	0.579	0.689	4 (rs1800469, VNTR, rs2227956, rs1801197)
Naive Bayes	0.569	0	0.620	3 (rs1800469, rs1800247, rs1801197)
Logistic regression	0.620	0.407	0.623	8 (rs1800469, rs1800629, rs6254, rs6256, rs2227956, rs1061581, rs1801197, rs17563)

AUC: area under the ROC curve.
